# Digital storytelling intervention to enhance social connections and participation for people with mild cognitive impairment: a research protocol

**DOI:** 10.3389/fpsyt.2023.1217323

**Published:** 2023-10-11

**Authors:** Di Zhu, Abdullah Al Mahmud, Wei Liu

**Affiliations:** ^1^School of Design and Architecture, Swinburne University of Technology, Hawthorn, VIC, Australia; ^2^Beijing Key Laboratory of Applied Experimental Psychology, National Demonstration Center for Experimental Psychology Education (Beijing Normal University), Faculty of Psychology, Beijing Normal University, Beijing, China

**Keywords:** co-design, mild cognitive impairment, digital storytelling, social connection, social participation

## Abstract

**Introduction:**

Interventions utilizing the principles of digital storytelling can improve cognitive ability by cultivating positive emotions and framing a new way to enhance social participation among people with mild cognitive impairment. However, existing research has understudied group-based storytelling, focusing instead on building individual stories and connections with family and friends. In response to this research gap, this paper proposes co-designing a digital storytelling intervention for people with Mild Cognitive Impairment (MCI) to enhance their social participation and build meaningful connections.

**Methods:**

We will conduct two co-design workshops with people with MCI (*n* = 12), their caregivers (*n* = 4–12), and therapists (*n* = 5) in Beijing, China, to facilitate the co-development of the digital storytelling application. During the first workshop, we will utilize card sorting and voting to define potential facilitators of social participation, identifying the abilities people with MCI want to improve through storytelling. During the second workshop, we will build on these findings to facilitate people with MCI and their caregivers to visualize the interfaces. After reflexive thematic analysis of the co-design workshops, we will develop a digital storytelling application and test its usability and efficacy among people with MCI and therapists, respectively. A single-blinded field test will be conducted with 20 community-dwelling adults with MCI (Age: 65+). The testing will consist of an intervention group of 10 participants who use the co-designed digital storytelling intervention and a control group of 10 participants who will not use the co-designed intervention on the waiting list. The intervention period will extend over 7 weeks, with individual intervention sessions lasting 30 min. We will evaluate its efficacy in terms of social participation, social connectedness, self-efficacy, subjective sense of happiness, and user experience of people with MCI.

**Discussion:**

This study will examine an innovative digital storytelling intervention to enhance social participation among people with MCI. This study is expected to advance the concept of community-centric social groups in social health service contexts by integrating technological solutions with the self-identified needs and lived experiences of people with MCI, increasing the motivation of people with MCI to cultivate social participation.

**Ethics and dissemination:**

Swinburne University of Technology’s Human Research Ethics Committee gave ethics approval for this research (Approval Number: 20226525-11105; Date: 26/09/2022). Our findings shall be reported in peer-reviewed journal articles and at relevant conferences.

## 1. Introduction

Mild Cognitive Impairment (MCI) is defined as an objectively identifiable cognitive impairment that is not severe enough to warrant a diagnosis of early dementia or major functional impairment, but which nonetheless results in subtle deficiencies in cogitative ability, often observed by the subject/patient or a relative (1, 2). Diminished social participation risks further cognitive decline (3) and social isolation (4). People with MCI may experience challenges from attention, memory, and executive function difficulties, as well as fatigue and decreased efficiency overall, with difficulties performing both familiar and novel activities in daily life (5). These factors impede social participation in social activities. MCI and dementia are interlinked, with a transition from mild impairment to dementia of 10–15% annually, reaching 50% within 5 years (6). Accordingly, to avoid MCI and, ultimately, the onset of dementia, interventions in low-and middle-income countries that increase social participation rates for people with MCI can be integral to improving their quality of life (7). However, people with MCI may experience limited cognitive abilities, behavioral challenges, and emotional changes during social participation, reducing their ability (or willingness) to function socially (8). In this context, several interventions have been developed to improve social participation, as described below.

### 1.1. Interventions to support social participation

Effective treatments, such as cognitive training (9), storytelling workshops (10), and cognitive behavior therapy emphasizing social skills (11) have been developed to encourage social participation for people with cognitive impairment. Some researchers focused on providing social interactions, such as new activities (12), or establishing social support groups (13). Most interventions for people with MCI often aim to improve cognitive abilities (14), but do not create new opportunities to build meaningful social connections, even a new form of social participation. Moreover, the approaches to improving the social connection of people with MCI have yet to be comprehensively investigated, despite social networks being found to be a significant mediator between MCI and mood issues in regression studies (15). Regarding modes of communication, some evidence suggests that regular phone-based communication with friends and family protects against MCI/dementia more than in-person meetings; however, a sample of people with MCI reported having as few as 0–1 phone conversation per week (16). People with MCI also report that they rely on increasing levels of support from close relatives and family members (17). Therefore, it is necessary to further investigate how their social networks can be improved vis-à-vis the experiences and clinical or community-based treatments of people with MCI.

Interventions incorporating storytelling therapies show promise in simultaneously improving cognitive, emotional, and social behavior. For instance, storytelling interventions have been shown to improve cognitive function, whether its stimuli were static images or virtual reality panoramas (18). People with mild to moderate dementia reported feeling much less emotional distress, anxiety, and despair (19), with research suggesting that a storytelling program has a story sharing via the program could enhance social connections through actively sharing stories with others (20–22). Moreover, digital technologies facilitate the production of enduring stories that can be readily disseminated to friends, family, and the general public (23). Digital storytelling systems created to connect people with dementia to their history and to enhance memories, communication, and social engagement have been greatly motivated by positive therapeutic outcomes (24). Technology-based applications are also beneficial for accessing rich and engaging multimedia recollection materials, enabling people with MCI/dementia to engage in media discovery and social contact, thereby taking charge of conversations (25). Concordantly, digital storytelling-based interventions show promise for improving cognitive ability, encouraging positive emotions, and framing a new way to enhance social connection and participation.

### 1.2. Complexity of needs in people with MCI of storytelling

Storytelling includes narrative storytelling and creative storytelling. The former aims to review and present stories they experienced before (26), and the latter aims to create a brand-new story with imagination (27). The creative storytelling program for people with MCI comprises five steps for presenting a picture: topic selection, background scenery, story development, character actions, and story ending (28). When compared to creative expression, narrative storytelling offers the advantage of customization, allowing the theme and plot to be tailored according to the interests and experiences of people with MCI, facilitating their active social participation. The linear structure of narrative storytelling aids in establishing a more organized and coherent story framework, alleviating the burden on memory. Therefore, this study limits the scope of narrative storytelling to support people with MCI. According to Rios Rincon’s review in 2022, most digital storytelling applications are designed to create individual stories rather than offering any group or collaborative format (workshops, classes, and courses) (29). In dementia care contexts, storytelling often refers to informal activities (30). In the individual application format, people with MCI counterintuitively have few social interactions with others, limiting the social impact (and, by extension, cognitive engagement) of the storytelling application. However, sharing stories with family and friends has been shown to enhance social connection (19). For instance, a board game, “This is Me” is designed to engage people with dementia to share life stories with their families and friends; they can recall stories based on cues on the cards (31). However, social participation emphasizes interaction outside one’s immediate circle of family and friends (32). To maximize cognitive engagement and social participation, digital storytelling applications should generate more interaction opportunities between unfamiliar friends and even strangers during story creation.

When people with MCI can access video or motion pictures of their stories, such as through digitization of old photographs or images and video taken on contemporary digital devices, they have the potential to share their stories with more people, including other group members or the public online. Some researchers have proposed online intergenerational digital storytelling applications whereby younger generations can post comments on the online stories of people with MCI (22, 33). Yet despite the therapeutic benefits, when sharing stories with strangers online, scholars in digital storytelling have stated the need for caution when sharing tales that could expose participants to potential injury and unfavorable reactions if shared with a broader audience (34). Accordingly, it is necessary to prioritize safety when building social interactions with more people.

Concerns regarding difficulties related to technology use remain an unsolved issue. People with MCI, on average, have lower digital literacy (35). Most storytelling programs include training sessions before the intervention, such as storytelling classes (20). Since it is primarily commercial off-the-shelf technology that is employed to support digital storytelling (29), these programs require some level of digital literacy in multimedia editing and storytelling abilities. Some studies asked trained volunteers (36) or their caregivers (37, 38) to support dementia patients in developing stories, such as drafting a storyline or editing a short video. However, these settings are designed to enable people with MCI to adapt to prevailing operating environments and interaction designs rather than customizing the software based on their personal needs, which would enhance usability while reducing learning costs. It is thus essential to further investigate how to design a user-friendly application to manage the resources and reduce the learning cost for people with MCI.

### 1.3. Co-designing digital storytelling applications

An individualized intervention focuses on a person’s needs, emphasizing improvements in cognitive abilities related to daily performance (39). The implemented application should function efficiently, safely, and reliably, placing user (and stakeholder) experiences at the center of the program (40). These criteria are best met by co-design, which necessitates a collaborative approach to design work. The designers play the role of facilitator, representing a shift from the traditional role of the translator (41). Co-design thus aims to provide an equal, respectful, and convivial manner, analyzing the use, design, and participation, and its key elements include creative contribution, equal collaboration, communication, and sharing ownership (42). Therefore, this study shall incorporate co-design principles in developing a digital storytelling application.

Participation throughout all stages of the development process is found to be crucial in the development of useful, friendly, supportive information technology (IT) applications that improve the quality of life for individuals with MCI (43), with prior experiences demonstrating the value of involving people with MCI in the design process (44). Participation has generally concentrated on the explorative and technical development phase (43). However, a few studies have reported involving people with dementia throughout all development sessions (45). Most prototype testing following the initial design involved people with MCI (46). As interest in co-designing with people with dementia continues to grow, some guidelines have emerged when conducting co-design sessions (47). Nonetheless, very little research directly involves people with MCI or clinical/therapeutic practitioners (48), with very little research recruiting early-stage dementia patients (49). In addressing this research gap, this study aims to enable people with MCI to engage in co-design sessions to express their feelings and ideas, creating a collective, entertaining design context wherein they can apply their knowledge.

Instead of co-designing a storytelling application involving people with cognitive impairment, more researchers aim to create their stories together (22, 33, 50). Our current understanding is thus guided by qualitative explorations of people with MCI’s experiences focusing on the needs of older adults, older adults suffering from loneliness, or people with dementia. Moreover, recent studies are beginning to focus on quantitative research designs to measure the effect of the intervention (51, 52). However, these neglect the importance of the qualitative research aims and its explanatory value in investigating unmet needs in the intervention context, such as the perceived usefulness of various daily-care activities (53), adherence support strategies (54), and goal setting (55). Therefore, it is necessary to further explore the unmet needs of people with MCI and identify how to enhance the storytelling experience by integrating technologies, linking to social groups, and designing adherence strategies.

### 1.4. Study aims

This study aims to enable people with MCI, caregivers, and therapists in collective and entertaining design moments to tailor a digital storytelling intervention conducive to cultivating social connection and participation. The program will identify the details of the digital storytelling intervention by proposing features, interactions, workflow, and interface elements.

- How can we enhance social interaction in creating storytelling in a group?
- How can we share stories using a digital platform in a community setting?
- How will the digital storytelling program be experienced by people with MCI and therapists?

## 2. Methods and analysis

Swinburne University of Technology’s Human Research Ethics Committee provided approval for this research (Approval Number: 20226525–11105, Date: 26/09/2022).

### 2.1. Study design

**Figure 1:**
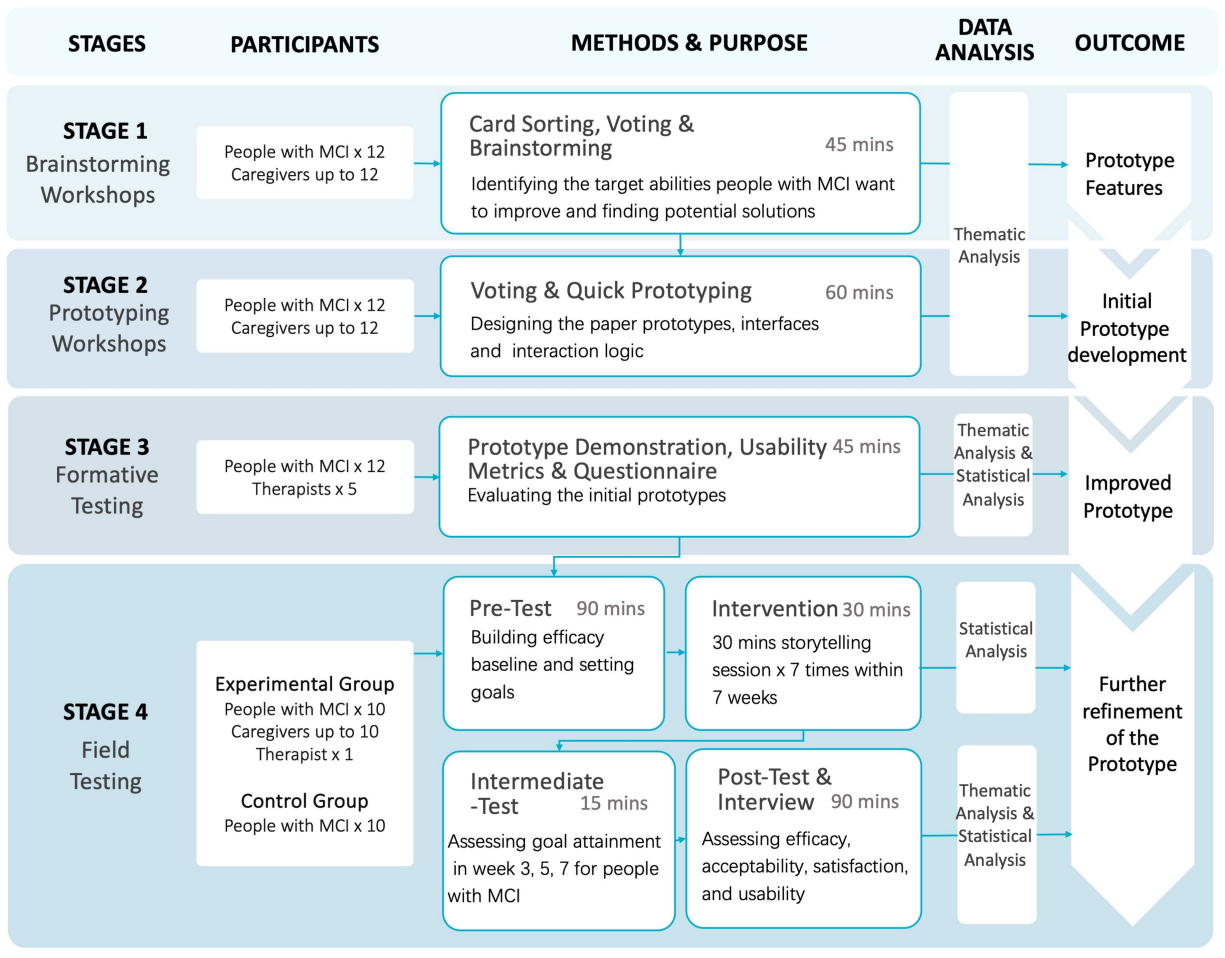
Overview of the research process.

Figure 1 illustrates the four sequential stages of this study: brainstorming workshops (Stage 1), prototyping workshops (Stage 2), formative testing (Stage 3), and field testing (Stage 4). At Stage 1, we shall identify the targeted abilities, thereby drafting intervention ideas. At Stage 2, we shall focus on designing and visualizing ideas. During Stage 3, we will assess the usability of prototypes through formative testing. Based on this feedback, we shall redesign the technology-based application. By Stage 4, we will evaluate the efficacy of the digital storytelling intervention. Each research phase will be conducted after all data collection and analysis of the foregoing research phase is completed. People with MCI included in the first three stages remain consistent. During the final stage, new people with MCI will be recruited to ensure accuracy.

On November 5, 2022, we conducted pilot studies for stage 1 and stage 2 involving four people with MCI and one caregiver. The sessions lasted approximately 45 min, during which we identified targeted abilities and potential features. Each person with MCI actively participated in brainstorming solutions to enhance their abilities with the assistance of facilitators. Stage 2, conducted individually, required 45–60 min per participant. Based on the diverse and abstract nature of the solutions identified during the pilot study, we revised the procedure to conduct stage 1 and stage 2 on different days with the same participants. We will analyze the results from stage 1 to determine potential intervention directions, followed by stage 2 to further refine and narrow down the ideas.

Stage 1 is projected to span 2 months. Following the result analysis, Stage 2 is estimated at 1 month, and an additional month will be allocated for mock-up development. Stage 3 is also anticipated to last 1 month. Our objective is to conclude data analysis and prototype development within a 2-month timeframe. The final stage is anticipated to require 3 months for completion.

We build upon the proposed co-design process by Sanders and Stappers (41), which includes three approaches: toolkits, probes, and prototyping. Co-design process would support us in better identifying the potential challenges that may limit the storytelling experience. Robinson et al. (56) proposed a co-design method to better explore the needs of people with dementia and their caregivers when using assistive technologies. Therefore, we adopt the co-design stages, comprising three main steps: a scoping stage, participatory design workshops, and prototype development. Moreover, we introduce the post-workshop stage (57): formative usability testing and field testing after developing the prototype.

Intervention idea brainstorming workshops will be organized based on the behavior change wheel (58), so as to design a social participation-centric behavioral change intervention. We selected the education, persuasion, incentivization, training, modeling, and enablement stages to frame the storytelling application. In the prototype development stage, we will utilize the prototyping design workshop to identify the intervention’s features, layout, and workflow. These two workshops involve people with MCI and their caregivers, working collaboratively. In the formative testing session, we shall involve therapists and people with MCI to conduct formative testing of the digital prototypes of the intervention, individually, circulating a short version User Experience Questionnaire to test attractiveness, perspicuity, efficiency, dependability, stimulation, and novelty (59, 60). The success rate shall be determined, and a five-point Likert scale utilized to test satisfaction. After formative testing, we shall summaries all feedback and suggestions, incorporating these in subsequent prototype improvements. Field testing will be conducted to evaluate the efficacy of the storytelling application, specifically the key measures of this study: the effects on social participation, social connectedness, self-efficacy, subjective sense of happiness, and user experience of people with MCI.

### 2.2. Participants

A social work organization (Jingshilaonian), in Beijing, China, has demonstrated a willingness to engage in this research. It is a psychosocial support institution located in Tiantongyuan Community, Beijing, which provides mental health services and daily activity support for older adults. The organization assisted us in the recruitment and screening of people with MCI. During the first three stages (see Figure 1), we will ask the psychology service to screen 12 people with MCI and distribute the information statement to people with MCI and their caregivers. We plan to conduct eight workshops with 12 people with MCI, and 4–12 caregivers, 17 participants to attend usability testing. We will collect about 8 h of group discussions and over 7 h of individual usability testing sessions. In our literature review of co-design studies on digital health solutions, we identified from the review paper by Sanz et al. (61) that nine studies employed workshops, with 77.8% of these studies involving fewer than 10 participants. Moreover, the sample size is consistent with established co-design research practices with people with dementia; for instance, one study used 10 participants and one caregiver (62); and another study used 6 participants (63). We will then invite five therapists to take part in the study. In the fourth stage, we shall recruit 20 people with MCI; 10 people with MCI will participate in the experimental study, the other 10 in the control group (on the waiting list), up to 20 caregivers, and one therapist. People with MCI will be allocated randomly into two groups. We will evaluate the performance of social participation of two groups and ask participants of people with MCI to join in our intervention after 2 months. Participants will be asked to complete the scales within a week.

The research process follows the willingness of people with MCI, who ultimately decide their level of involvement in the research sessions (64). For instance, they can draw, show, or describe the expected interfaces. When “dementia time” of people with MCI occurs, the caregiver shall decide if it is possible to continue the research or to seek the therapist’s support. This study shall document communication and usability issues encountered during testing, with results serving as an important basis for design iteration (65).

Inclusion criteria for people with MCI are those who are clinically diagnosed with MCI and live in community-dwelling contexts (live independently in their own homes or apartments within their local communities, as opposed to residing in institutional care settings.) in Beijing, who are aged above 65 years old and who do not have a visual or hearing impairment, and who have sufficient reading ability to engage with the interface. In addition, the participants must agree on informed consent and have a formal or informal caregiver who is also motivated to participate, as caregivers shall provide feedback and score the goal performance. Exclusion criteria for people with MCI include having experienced a significant neurological condition such as stroke or brain injury, since such conditions would impact daily functioning and may serve as a potential confounding factor. There are no specific inclusion criteria for caregivers. Inclusion criteria for therapists require them to possess a postgraduate degree or above and to have working experience in social interventions for over 3 years. Therapists with an experience of more than 3 years are being sought due to their potential to offer more valuable insights and suggestions. Their extensive experience equips them with a deeper understanding of the needs of people with MCI and their family members, making their input particularly valuable. Therapists unwilling to sign the informed consent form shall be excluded.

### 2.3. Workshop procedures

#### 2.3.1. Stage 1: brainstorming workshops

In stage 1, we will organize four workshops involving people with MCI and their caregivers to explore the most compelling benefits, role models, and barriers they prioritize in achieving significant social participation. It will support people with MCI to understand the importance of social participation and enhance the data quality of stage 2. Each workshop will vote on one of the most compelling benefits, role models, and one of the people with MCI will be asked to select two of the abilities they want to improve. In the subsequent sections, we will present the outcomes of these workshops and elucidate the rationale behind their choices. It lasts 45 min, comprising three sub-stages: (1) be an advisor; (2) abilities card sorting; (3) solution brainstorming (please refer to Supplementary Appendix 1).

##### 2.3.1.1. Phase 1: be an advisor-decision making (25 min)

We will provide existing benefits to participating people with MCI and their caregivers. Core beliefs represent the concepts of cognitive behavior therapy, a mental support therapy method employed to treat many common mental health problems. Three people with MCI (with their caregivers) will be allocated to the same group. First, the group will be asked to select the most important benefits of attending social activities from an arbitrary list provided. The facilitator shall ask them why these specific benefits are chosen and how they will introduce the benefits and persuade other people with MCI to join social activities. Participants shall be asked to select the most important reward from a pre-populated list we provide. We then will ask participants which role model inspires them to engage in social participation.

##### 2.3.1.2. Phase 2: abilities card sorting (10 min)

To improve social participation, we will ask which abilities or skills people with MCI wish to improve, specifically asking each person with MCI to sort these nine abilities into three categories: must improve, could improve, and no need to improve. Based on their selection, we will analyze identify the most important ability for improving social participation.

##### 2.3.1.3. Phase 3: solution brainstorming (10 min)

To further improve social participation, we will ask which abilities people with MCI want to improve. Each participant will be asked to select two abilities and to give their reasons. For example, they could select their memory ability or language expression ability. Based on their selection and stories, we will summarize the potential features of the storytelling application. We will then use this information to provide potential solution lists, which shall be discussed by the research team and a psychologist specializing in aging.

#### 2.3.2. Stage 2: prototyping workshops

During the prototyping session, three people with MCI (with their caregivers) will be asked to build prototypes on paper, supported by the researchers to define interaction logic and scenarios. This session shall comprise three stages: (1) storytelling application brainstorming, (2) designing features, and (3) storytelling. Participants will remain in the same groups as in Stage 1. The storytelling application aims to combine self-reminiscing (preparing materials with cues) and group reminiscing (discussing with group members regarding a certain topic) to support people with MCI in preparing their stories and thereby enhancing group interaction, social connections, and social participation.

##### 2.3.2.1. Phase 1: features brainstorming of storytelling application (10 min)

Based on the findings of Stage 1, we now focus on designing a digital storytelling application. The facilitator will continue to support people with MCI in discussing how the application can support storytelling in a group setting, meet their expectations, and encourage them to suggest features. The facilitator will ask people with MCI and their caregivers to identify the interaction of application and storytelling themes they are interested in.

##### 2.3.2.2. Phase 2: designing features (40 min)

Storytelling applications are primarily supported by technologies in story creation and story sharing. The MESSAGE program (see Table 1) is proposed in order to maximize successful communication between people with dementia and their caregiver (66). The program has been evaluated among community-based aged care staff caring for dementia patients. It has been demonstrated that caregivers report significant increases in knowledge and readiness to care for people with dementia after receiving communication training. The MESSAGE program could thus be utilized to facilitate communication between people with MCI and smart devices. Therefore, before design sessions, we utilized MESSAGE communication strategies to support communication throughout the digital storytelling process. Since we aim to propose detailed features, we converted the strategies into an application and shall follow these strategies to ask in-depth questions to people with MCI, in order to identify effective and useful interfaces and interactions.

**Table 1:** MESSAGE communication strategies and design guidelines transition.

MESSAGE Communication Strategy	Adapted Strategy	Explanation
M-MAXIMIZE attention	M-MAXIMIZE attention	Attract group attention One speaker at a time Avoid distractions
E-Watch your EXPRESSION and body language	E-ENHANCE the group interaction	Create opportunities to interact with group members Facilitate group discussions
S-Keep it SIMPLE	S-Keep the interaction SIMPLE	Use short, simple, familiar content to design interfaces Give clear options to advance to the next step
S-SUPPORT their conversation	S-SUPPORT their reminiscing	Provide sufficient time Remind participants of the topic Provide an equal chance to reminisce
A-ASSIST with visual AIDS	A-ASSIST with reminiscing cues	Related materials of the topic Show the cues to support reminiscing
G-GET their message	G-GET prepared in advance	Prepare individual materials in advance Ensure familiarity with these materials Add notes to the materials
E-ENCOURAGE and ENGAGE in communication	E-ENCOURAGE and ENGAGE in communication	Discuss interesting and familiar topics Talk about individual experiences

Participants shall be asked to individually discuss the features identified in Phase 1 and consider the sequences of interfaces and interactions. The facilitator shall lead a group of people with MCI to discuss the main features, including generating materials, reminiscing, sharing, and training. During the discussion, participants shall be invited to propose the content with text, images, or video of the application. Each feature will be visualized by a participant who uses a printed phone/tablet/website template to sketch the content and layout of the interface, with the support of the facilitator.

##### 2.3.2.3. Phase 3: storytelling (10 min)

We shall lead people with MCI to review the full scope of interaction and ensure the storytelling application is holistic and complete. We then will ask people with MCI to summarize how they will use the application. The facilitator will check and ask people with MCI if it could meet their needs, taking this as an opportunity to identify other features to add. Finally, the facilitator will ask people with MCI to brainstorm when older adults would use the application in daily life.

#### 2.3.3. Stage 3: formative testing

Building upon the findings of the previous stage, we will analyze the features of the prototypes and design the initial prototypes. This stage seeks to identify and resolve usability issues. Participants will test the initial prototype separately (35 min), giving feedback via a structured interview (20 min).

##### 2.3.3.1. Phase 1: usability testing (35 min)

Participants shall first engage in usability testing and are asked to complete a set of tasks, including filling out the UEQ (User Experience Questionnaire – Short Chinese Version) to ascertain the usability level of the technological intervention program (59). The mock-up will be presented to participants for feedback on content and design.

##### 2.3.3.2. Phase 2: structured interview (20 min)

As our primary goal is to collect feedback from people with MCI, our objective revolves around acquiring a comprehensive view of the designed mock-ups. For this purpose, we have crafted 12 close-ended questions to capture an overarching impression. Following usability testing, we will then conduct structured interviews (see Supplementary Appendix 2), asking respondents to select the most preferred and most disliked mock-ups.

#### 2.3.4. Stage 4: field testing

After analyzing the formative testing results, we will then revise the initial prototypes based on the identified usability issues and user suggestions. This stage aims to identify the limited efficacy, including social participation, social connectedness, self-efficacy, subjective sense of happiness, and user experience. We shall recruit 20 people with MCI; 10 people with MCI will be allocated to the experimental group. Field testing comprises three main phases (see Table 2 for the timeline for field testing). The pre-test shall take 90 min. We prefer that participants complete the scales on the day immediately following the initial test to minimize potential fatigue-related effects. However, recognizing that participants may find it challenging due to fatigue or other factors, we will also allow the option of completing the scales on the subsequent day. Building upon technology–based intervention testing literature, we shall conduct 2 or 3 months of testing (67–69). Therefore, people with MCI shall be asked to participate in 30-min interventions per week, totaling seven sessions. Every 2 weeks, we will conduct a 15-min online goal attainment meeting. The post-test shall take 60 min. Interviews with people with MCI, caregivers, and therapists will take 30 min, 15 min, and 60 min, respectively.

**Table 2:** Timeline for the field testing.

	Enrolment	Allocation	Post-allocation								Close-out
Timepoint (weeks)	−2	0	1	2	3	4	5	6	7	8	9–10
Intervention: in waiting list (control)	Enrolment and informed consent: eligibility screen (MoCA-C)	Pre-test									Post-test and interview
Storytelling application use (experimental)		Pre-test	Training	Intervention 1	Intervention 2	Intervention 3	Intervention 4	Intervention 5	Intervention 6	Intervention 7	Post-test and interview
Intermediate measures (goal attainment)					Follow-up 1		Follow-up 2		Follow-up 3		

##### 2.3.4.1. Phase 1: pre-test: baseline building and goal setting (90 min)

Participants shall be asked to fill in a paper copy of the self-report questionnaire, supported by the researchers. In order to capture baseline data from people with MCI, we include social participation [LIFE-H 77 items (70)], social connectedness [SCS 20 items, see Lee and Robbins (71)], self-efficacy [GSES 10 items, see Zeng et al. (72)], subjective sense of happiness [SWS 20 items, see Diener and Emmons (73)]—all of which are provided in Chinese. After testing, we will present the program to the participants, teach them how to use it, and provide them with a brochure in case they forget the procedure. Before the formal intervention, people with MCI will be invited to join in the training sessions to become familiar with the program, so that they can use the program autonomously (74). Except for the evaluation of cognitive function, this study will evaluate the social features of participants, including motivation. It will utilize four validated instruments to test social participation, social connection, self-efficacy, and subjective sense of happiness (see Data Collection section). The therapists will use the programs to co-establish the intervention goals in the first week. After seven interventions, we will ask people with MCI to answer the User Experience Questionnaire [UEQ 8-item, Chinese version, see Schrepp et al. (59)] to identify user experiences vis-à-vis the application.

##### 2.3.4.2. Phase 2: intervention (30 min/7 times) and intermediate test for people with MCI: goal attainment (15 min/4 times)

We will provide the technology-based intervention to people with MCI, asking them to complete seven sessions over a two-month period, with each session lasting 30 min. During the intermediate tests, we will follow the goal-setting approach of Clare et al. (75) to collect goal attainment, problems, suggestions, or general comments every 2 weeks.

##### 2.3.4.3. Phase 3-a: post-test for people with MCI: assess efficacy, acceptability, satisfaction, and usability (60 min)

Incorporating the findings of Kitakoshi et al. (76), this study will utilize seven validated instruments and one goal attainment from Clare et al. (77) measuring the improvements of people with MCI. After a two-month test, we will use instruments to test, including social participation, social connection, self-efficacy, subjective well-being, motivation, acceptability, satisfaction, and usability (UEQ 8 items).

##### 2.3.4.4. Phase 3-b: interviews with people with MCI (30 min), caregivers (15 min), and therapists (60 min)

At the end of the intervention, we shall conduct interviews separately, investigating people with MCI’s social participation and self-management changes, collecting suggestions about the intervention and the program. We also take this opportunity to follow up on notes and ask questions to understand their individual difficulties. Conversation is recorded, enabling us to analyze feedback and improve prototypes. We shall ask for goal attainment, problems, suggestions, or general comments at the end of the intervention.

### 2.4. Data collection

The study shall collect audio recordings and photographs of sketches during the co-design workshops, supplemented by questionnaire responses. Participants shall be informed of the audio recording and photography before the workshop, and consent shall be obtained. Qualitative data shall be collected from the co-design study. Quantitative data shall also be collected from the formative testing and field testing. We use the assessment of life habits (LIFE-H) to measure social participation, Social Connectedness Scale (SCS) to measure social connection, General Self-Efficacy Scale (GSES) to measure self-efficacy, Subjective Well-Being Scale (SWS) to measure subjective sense of happiness, and User Experience Questionnaire (UEQ) to measure user experience. As shown in Table 3, all instruments employed in this study have already undergone psychological validation.

**Table 3:** Measurement and instruments for people with MCI.

Measure	Instrument	Items
Social participation	The assessment of life habits (LIFE-H)	77 items
Social connection	Social connectedness scale (SCS)	20 items
Subjective sense of happiness	Subjective well-being scale (SWS)	20 items
Self-efficacy	General self-efficacy scale (GSES)	10 items
User experience	User experience questionnaire (UEQ)	8 items

#### 2.4.1. The assessment of LIFE habits

This questionnaire investigates 12 categories of life habits that ascertain daily activities and social roles (with limitations) for old adults (70). All statements present the social participation profile of the respondent by measuring the quality of social participation and level of satisfaction. This study employs the short Mandarin version 3.0 with 77 items, since the short version is the only available Mandarin translation. LIFE-H is a proven reliable instrument that focuses on understanding concepts of functional or function independence (78), helping to indicate the impact of behavioral strategies on older adults (79).

#### 2.4.2. Social connections scale

Developed by Lee and Robbins (71), SCS is the most commonly used international instrument for measuring social connectedness. A self-assessment scale, the SCS is unidimensional with eight questions that are all negative expressions, which remains its biggest drawback. In order to avoid the negative impact of this, Lee modified the more extreme representations of the SCS by adding two negative and 10 positive items to create a 20-item SCS-R (80). The Chinese version of the scale, introduced by Fan et al. (81), demonstrated good reliability with α coefficient of 0.92 for the overall scale, 0.82 for the socially connected Cronbach’s coefficient, and 0.87 for the socially unconnected Cronbach’s coefficient. The Chinese version contains 20 items; each item is scored on a 6-point Likert scale, with 1 to 6 indicating a range from “strongly disagree” to “strongly agree”; the higher the score, the higher the level of social connectedness.

#### 2.4.3. Subjective well-being scale

Developed by Diener and Emmons (73), the SWB is a holistic assessment of an individual’s quality of life based on self-referential criteria and contains two dimensions: affective and cognitive (82). The Chinese version has subsequently been revised and contains 20 items. A 5-point Likert scale is used to assess the degree of feelings, reactions, and recognition of the participant. Questions 4, 5, 6, 9, 10, 11, 13, 15, 17, 18, and 20 are reverse scored (83). The internal consistency coefficient was 0.8475, with good homogeneous reliability. The scale boasts good criterion validity and conceptual validity.

#### 2.4.4. General self-efficacy scale

Self-efficacy refers to one’s self-beliefs in the particular context of functioning. It is the most common scale used to evaluate people’s perceived self-efficacy. GSES focus on action self-efficacy and coping self-efficacy, via 10 items, aiming to ascertain a general sense of personal competence to deal daily challenges across various contexts (84). It has been demonstrated that GSES is conducive for researchers investigating self-efficacy in Chinese contexts, Zeng et al. (72) translated it from traditional Chinese to simplified Chinese, showing good internal consistency (α = 0.91).

#### 2.4.5. User experience questionnaires

UEQ is a simple, reliable, and demonstrably valid approach for adding a subjective quality assessment to the data from other evaluations, which has validated the Chinese Version (59). The short version of the questionnaire captures a comprehensive impression of user experience. Both classical usability aspects (efficiency, perspicuity, dependability) and user experience aspects (originality, stimulation) are measured (60). There are no costs and no restrictions when using the UEQ questionnaire in medical informatics applications (85), making it ideal for our purposes of measuring the user experience of digital storytelling intervention.

#### 2.4.6. Goal attainment

Goal attainment combines the five times (four times during the intermediate intervention, one time at the end of the intervention) goal attainment from the self-report, and the evaluation from their caregivers and therapist (75).

### 2.5. Data analysis

Quality data analysis from workshops and interviews shall follow the thematic analysis (86, 87). Since through thematic analysis, we can discover hidden user needs, behavioral patterns, and trends in co-design data, and they can be extracted to provide important clues for further design digital interventions (42). We will select the transcript with the richest information to summarize the codebook with code and definition. Two researchers will review and code the transcripts based on the codebook. Both researchers will independently code all the transcripts. To enhance the reliability of the analysis, we will use NVivo to calculate inter-coder reliability (88). Any discrepancies in coding will be resolved through collaborative discussions to ensure consistent interpretation and coding. After which, the codes shall be reorganized into themes. NVivo will be used during the analysis stage. We will analyze the targeted abilities, reasons, and potential intervention directions to enhance social participation. In stage 2, we will extract feature descriptions of prototypes and sketches of interactions. Subsequently, we will categorize this data into distinct features with corresponding explanations, and interactions. We will build the mock-ups following the feature clusters. At stage 3, we will cluster usability issues and suggestions. At the last stage, we will analyze the suggestions.

Quantitative data includes questionnaire data. The efficacy evaluation utilizes post-test data, including baseline data and potential confounders as covariates, so as to test differences between the groups on the primary outcome measures. Following the completion of data collection, intention-to-treat and “per protocol” analyzes shall be undertaken. When research participants withdraw (thereby leaving us with missing data), we shall apply appropriate techniques to impute that data and conduct sensitivity analyzes as necessary. SPSS will be employed for the analyzes. In addition to *p*-values, we additionally publish effect sizes with computed 95% confidence ranges. We also employ 0.05 alpha 2-tailed *p*-values for the efficacy of improving social participation.

### 2.6. Data management and monitoring

The hard copy questionnaires shall not contain any participant-identifying information. All completed hard copy forms and informed consent forms shall be kept in a secure facility behind locked doors. Regarding the quantitative data collected, descriptive statistical analysis (means and standard deviations) shall be conducted. The audio recordings of the workshops will be transferred to a local password-secure computer for storage, and the recording device will be wiped and removed. The local computer will be accessible only to the investigators. All records and audio recordings will be kept for a maximum of 5 years following the completion of this study and the publication of its findings. Any adverse events during the investigation shall be reported to Swinburne University of Technology’s Human Research and Ethics Committee, as per the institutional policy.

## 3. Discussion

This study examines an innovative digital storytelling intervention that aims to enhance social participation among people with MCI who live in low-income countries. Our focus is on designing a technology-based intervention that would post activities, support people with MCI in collecting materials, and support group reminiscence. Commercial applications in storytelling interventions almost always include application training sessions or other pre-use supports for people with MCI (50, 89). However, as identified above, the applicability of these methods for storytelling among MCI adults is presently not comprehensively understood (90). Concomitant with training to ensure a proper use process, it is necessary to further explore ways to enhance the understanding of user interface interaction among people with MCI. Since a fundamental understanding of digital applications necessitates the comprehension of e-skills, it guides the interaction rather than encouraging memorization of the interaction steps (91). It thus becomes necessary that we further investigate how best to design user-friendly applications to manage available resources and reduce learning costs for people with MCI most effectively.

Encouraging storytelling among more people with MCI promises to be an effective social activity in community-based care, since it enhances social interactions among a greater number of people. However, privacy issues risk manifesting when people with MCI seek to share stories with more people, especially in public online forums. Some researchers have posited that intergeneration digital storytelling applications could encourage younger generations to post comments and engage with the online content (22, 33). However, when sharing personal stories (specifically photographs and other media) with strangers online, scholars in digital storytelling are right to emphasize caution, especially when sharing tales that could expose participants to potential injury and unfavorable reactions (34).

Accordingly, it becomes necessary to identify ways that support people with MCI to safely build social interactions with more people, while concurrently identifying conducive storytelling topics for group storytelling or online sharing. To ensure more active social interactions, these topics should be attractive, interesting, and arousing in terms of memory; contrariwise, they should not be overly personal, boring, or arousing memories of difficult times. Moreover, the location of storytelling sessions may lead to different user groups, such as in care centers, local community groups or online groups. Different user groups exhibit different needs regarding their preferred topics, as well as variable interaction logic when using the storytelling application. This study aims to investigate the needs of the community-dwelling people with MCI, since they benefit from a higher digital literacy level, and exhibit greater motivation to attend social activities. The researchers intend to test the proposed approach in care centers and online communities. This study thus contributes to the literature by offering a new form of social, community-centric engagement by integrating technologies with people MCI’s therapies, increasing the motivation of people with MCI to enhance and cultivate their social participation.

Moreover, the designed digital storytelling intervention may benefit wider user groups, since by integrating social interaction with thematic interests and streamlining operations, we can create an engaging and meaningful digital story sharing platform for seniors and foster valuable social participation moments. This platform facilitates group-based digital storytelling, enabling people with MCI as well as those experiencing normal aging to forge connections and enrich their social networks through sharing and listening to others’ stories. Additionally, organizing offline meet-up events fosters face-to-face communication and sharing, further enhancing participation. Through these initiatives, normal aging older adults can relish interactive experiences, foster active involvement, and cultivate stronger social bonds. Simplified user interfaces, accompanied by clear guidance and support, ensure swift adoption and enjoyment of the digital story sharing process.

## Data availability statement

The original contributions presented in the study are included in the article/Supplementary material; further inquiries can be directed to the corresponding author.

## Ethics statement

Ethical approval has been granted by Swinburne University of Technology's Human Research Ethics Committee gave approval for this research (Approval number Ref: 20226525-11105, date: 26/09/2022). Written informed consent will be obtained from participants.

## Author contributions

AAM and DZ contributed to the conception and design of the study. DZ wrote the first draft of the manuscript. AAM and WL supervised the project and assisted in rewriting several sections of the manuscript. All authors contributed to the article and approved the submitted version.
