# Supplementary material for: Digital storytelling intervention to enhance social connections and participation for people with mild cognitive impairment: a research protocol
Source: Front Psychiatry. 2023 Oct 11;14:1217323. doi: 10.3389/fpsyt.2023.1217323 (PMC10599142; doi:10.3389/fpsyt.2023.1217323)
Supplement: Supplementary file 1 [file Data_Sheet_1.docx]

# Appendix

**Appendix 1:**

**Stage 1: Intervention Idea Brainstorming (60mins)**

1. **Be an advisor-decision making (25mins).**

- Questions:

Benefits

1. Please select the most important benefits of social participation.
2. Why do you think it is the most essential benefit of social participation? How will you introduce the benefits to others?

Wrong ideas

1. Please select the most common misunderstanding about social participation others may hold.
2. Why do you think it is the most urgent misunderstanding to overcome first? How will you break this misunderstanding?
3. How will you appeal to and persuade others to join social activities more?

Rewards

1. Here are the rewards for social participation; please select the three most important rewards and rank them.
2. Why do you think it is the most important? Do you have any stories to share?

Role models

1. Here are some role models that may inspire participants to engage in social participation. Please select the best example.
2. Why do you think it is the best example? Do you have any stories to share?

Table A 1 Benefits, wrong ideas, rewards, and role models list

| Category | List of options | Questions |
| --- | --- | --- |
| Benefits | - Improve physical fitness - Improve mental health - Cultivate your own interests - Learn new knowledge - Gain a sense of identity - Relieve stress - Realize self-value - Improve life happiness - Enrich spiritual life | - Please select the most important benefits about social participation. - Why do you think it is the most important benefits? How will you introduce the benefits to others? |
| Wrong idea | - Social participation is the expression of the elderly's "doing nothing" - Social participation means that the elderly "cannot stay at home" - Social participation is the expression of the elderly who "have no children to look after" - Social participation is the expression of "no serious business" of the elderly - Social participation costs energy and time and is a burden for the elderly | - Please select the most common misunderstanding about social participation others may hold. - Why do you think it is the most urgent misunderstanding to overcome first? How will you break this misunderstanding? - How will you appeal and persuade others to join social activities more? |
| Rewards | - Encouraged by peers - Encouraged by professionals - Encouraged by his family - Encouraged by the younger generation - Encouraged by friends - Increased confidence - Gained respect - Improved physical condition | - Here are the rewards for social participation. Please select the three most important rewards and rank them. - Why do you think it is the most important? Do you have any stories to share? |
| Role models | - A selfless person - An influential person - A confident person - An energetic person - An active exerciser | - Here are some role models may inspire participants to engage in social participation. Please select the best example. - Why do you think it is the best example? Do you have any stories to share? |


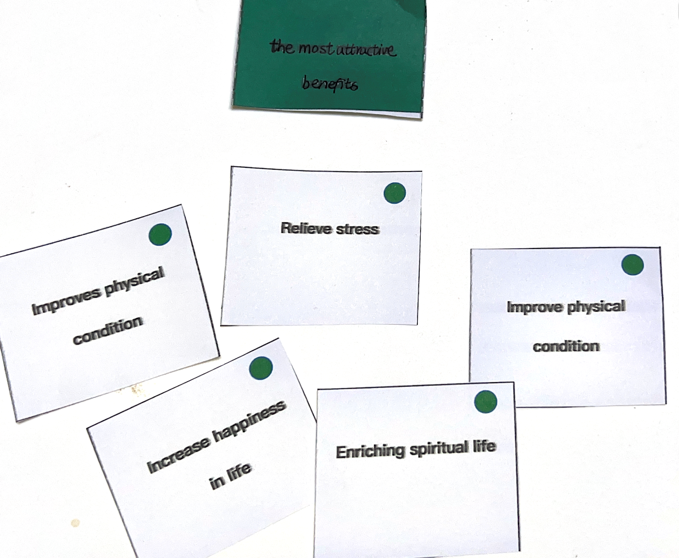


Figure A 1 Toolkit of “Be an advisor section”

1. **Abilities card sorting (10mins).** To improve social participation, we will ask which abilities or skills that people with MCI want to improve. We will ask every people with MCI to sort these nine abilities into MUST IMPROVE, COULD IMPROVE, NO NEED TO IMPROVE categories. Based on their selection, we will identify the most important ability.

Questions:

1. Here are some abilities helpful to your social participation. Please sort these nine abilities into MUST IMPROVE, COULD IMPROVE, NO NEED TO IMPROVE (Table 2 and Figure 2).
2. Please select the most urgent ability you want to improve in “MUST IMPROVE” cluster.
3. Why do you think they are important? Do you have any stories to share?


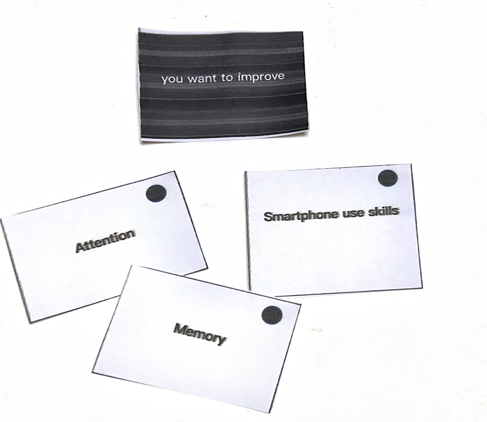

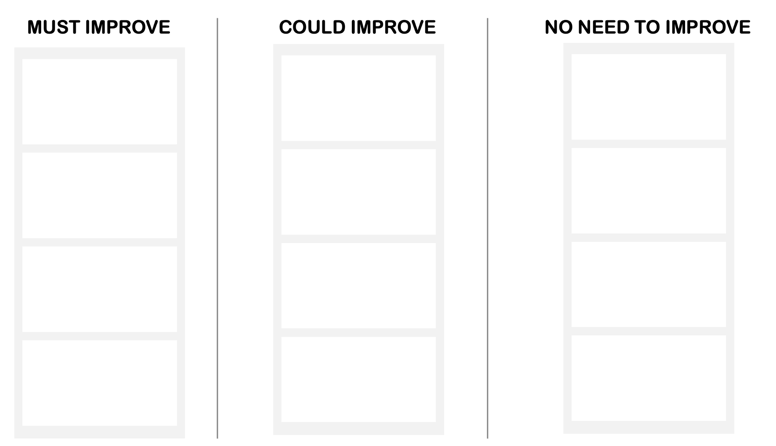


Figure A 2 Card sorting toolkit in “Ability card sorting section”

Table A 2 Abilities list of people with MCI may want to improve.

| Abilities List | Questions |
| --- | --- |
| - Concentration - Memory - Self-confidence - Language expression ability - Smartphone skills - Rapid recovery - Coping with stress - Communication skills with Family - Find your interests | - Here are some abilities are helpful to your social participation. Please sort these nine abilities into MUST IMPROVE, COULD IMPROVE, and NO NEED TO IMPROVE (Table A 2 and Figure A 2). - Please select the most urgent ability you want to improve in “MUST IMPROVE” cluster. - Why do you think they are important? Do you have any stories to share? |

1. **Solution brainstorming (10 mins).**

- Questions:

1. If you want to improve the selected ability, do you have some tricks or expectations to share? What effect do you want to achieve?
2. Here are some potential solutions (show the solutions from Table A3), which you want to include in future design.
3. Why do you think they are important? Do you have any stories to share?

Table A 3 Solutions, potential features for each ability

| Abilities | Solutions | Potential Features |
| --- | --- | --- |
| Attention | Get focused tricks | Display tricks, review tricks, summarize tricks |
|  | Attention training game | Rules of game, interface style, novice teaching, training activities, performance record |
|  | Meditation | Voice guidance, interface style, novice teaching, training activities, performance record |
| Memory | How to use to-do list | Novice teaching, feature practice, instruction query |
|  | Memory tricks | Display tricks, review tricks, summarize tricks |
|  | Memory training game | Rules of game, interface style, novice teaching, training activities, performance record |
| Enhance self-confidence | Building confidence tricks | Display tricks, review tricks, summarize tricks |
|  | Acquire more knowledge toward aging | Select lectures, attend lectures, take notes, review, summarize knowledge |
|  | Summarize recent achievements | Performance record, performance display |
| Verbal communication ability | Communication tricks | Display tricks, review tricks, summarize tricks |
|  | Reading aloud | Select document, reading aloud, review, save, share, performance record |
|  | Dubbing | Select video, dubbing, review, save, share, performance record |
| Smartphone use skills | Smartphone using lecture | Select lectures, attend lectures, take notes, review, summarize knowledge |
|  | Usage quiz | Select scope, do the quiz, review, summarize knowledge |
|  | Updated version function teaching | New feature practice, instruction query |
| Fast fatigue relief | Avoid fatigue tricks | Display tricks, review tricks, summarize tricks |
|  | Recommend appropriate activities according to physical condition | Do a test, get the suggested list, select at least one activity, plan |
|  | Meditation | Voice guidance, interface style, novice teaching, training activities, performance record |
| Coping with stress | Meditation | Voice guidance, interface style, novice teaching, training activities, performance record |
|  | Breathing exercises | Voice guidance, interface style, novice teaching, training activities, performance record |
|  | Mood diary | Novice teaching, feature practice, write down daily mood, save, summarize the mood, share |
| Communication skills with family | Communication tricks | Display tricks, review tricks, summarize tricks |
|  | Communication lectures | Select lectures, attend lectures, take notes, review, summarize knowledge |
|  | Contact your family regularly | Set a communication cycle, select contact, select way of reminding |
| Finding your own interests | Interests test | Do a test, get the suggested list, select at least one activity, plan |
|  | Recommend suitable activities according to personality traits | Do a test, get the suggested list, select at least one activity, plan |
|  | Suggestions for interest attempts | Get the suggested list, select at least one activity, plan |

1. **Break: 15mins**

**Stage 2: Rough prototyping workshop (60mins)**

**1. Storytelling application brainstorming (10 mins).**

Questions:

1. If you are asked to share your experience or stories with new friends, what would you like to share? Which topics do you want to discuss more? Please write each on post-it notes.
2. What is your expectation of the application to support group storytelling?
3. When you are telling a story, what type of information may help you to engage yourself to recall the memory?

**2. Design features (40 mins)**

Questions:

- - 1. When you are preparing your stories to share with new friends, do you need to take down the notes?
    2. How would you like to capture, edit, and upload it?
    3. When you are telling a story, what type of information may help you to engage yourself to recall the memory?
    4. How will the information help you to tell the stories?
    5. After your sharing with new friends, how could you encourage these new friends to discuss the topic with you?
    6. Do you wish you had a predetermined story frame (time, place, characters, etc.) to give you some hints in the process of creating the main threads of the story? How would you like it, and do you want the clues to appear randomly or in order?

**3. Storytelling (10 mins)**

Questions:

1. Please check the sequence of the features. Are there any new things you want to add?
2. Please tell us when you will use these tools or features in daily life.
3. Explain how you would like to integrate the application into your daily life? When will you use it?
4. Any other expectations or features you suggested?

**Appendix 2:**

Usability testing – semi-structure interviews

- What do you think about the applications?
- Do you find it easy to use?
- Do you think that the instructions given by the application are clear and easy to understand?
- Do you think it satisfies your needs?
- Do you feel this application can be of benefit to you?
- Which application do you like most?
- Which application do you like least about it?
- What do you like most function about the application? (advantages)
- What do you like the least (i.e., function) about it the application? (disadvantages)
- What should be improved?
- Have you met any difficulties?
- Would you like your family, friends, neighbors, etc., to use the program and recommend it to them?
